# Effect of Staggered vs. Simultaneous Co-Administration of Bempedoic Acid on Pharmacokinetics of Pravastatin: Randomized, Cross-Over Clinical Trial in Healthy Volunteers

**DOI:** 10.3390/pharmaceutics17010060

**Published:** 2025-01-03

**Authors:** Felicitas Stoll, Salvatore Amato, Max Sauter, Jürgen Burhenne, Johanna Weiss, Walter E. Haefeli, Antje Blank

**Affiliations:** 1 Medical Faculty Heidelberg, Heidelberg University, 69117 Heidelberg, Germanyantje.blank@med.uni-heidelberg.de (A.B.); 2 Internal Medicine IX—Department of Clinical Pharmacology and Pharmacoepidemiology, Heidelberg University Hospital, 69120 Heidelberg, Germany

**Keywords:** bempedoic acid, pravastatin, OATP1B1, SLCO1B1, inhibition, staggered administration, healthy volunteers, pharmacokinetics, drug interaction

## Abstract

**Background/Objectives**: Bempedoic acid (BA) is a novel cholesterol-lowering agent with proven positive effects on cardiovascular endpoints. Because it is an inhibitor of the hepatic transporters OATP1B1 and OATP1B3, two uptake transporters regulating the intrahepatic availability of statins, it increases the systemic exposure of co-administered statins. This interaction could raise the risk of myopathy. We hypothesized that the drug interaction between BA and statins could be mitigated by staggered administration. **Methods**: This was a single-centre, open-label, randomized, two-arm, cross-over, phase I drug interaction trial in healthy volunteers (EudraCT-No: 2022-001096-13). The primary objective was to evaluate the OATP1B1 inhibitory effect of BA on exposure to pravastatin after simultaneous administration versus different schedules of staggered administration. A secondary objective was to evaluate the impact of *SLCO1B1* genotypes (*1, *5, *15, *37) on pravastatin exposure. Pravastatin was administered in single oral doses of 40 mg at six visits. After a baseline visit with pravastatin alone, BA was dosed to steady state at the approved oral dose of 180 mg. Outcome measures were the area under the plasma concentration–time curve, extrapolated to infinity (AUC_∞_) and C_max_ of pravastatin, 3α-hydroxy-pravastatin (pravastatin 3-iso), and pravastatin lactone, and their geometric mean ratios (GMRs) of different schedules of administration. Log-transformed AUC_∞_ and C_max_ were compared with one-way ANOVA with a 90% confidence interval (CI). **Results**: Fourteen participants completed all visits. At BA steady state, the GMRs of pravastatin AUC_∞_ and C_max_ were 1.80 (90% CI 1.31–2.46) and 1.95 (90% CI 1.40–2.72), respectively, compared to baseline. There was no significant difference in pravastatin exposure between simultaneous vs. staggered administration. There was no statistically significant difference in pravastatin 3-iso or pravastatin lactone between different administration modes. For the AUC_∞_ of pravastatin and pravastatin 3-iso, haplotype was a significant source of variation (63% and 20%, respectively), while the type of administration (simultaneous vs. staggered) had no significant impact. **Conclusions**: The increase in pravastatin exposure with concomitant intake of BA was larger than expected. There was no significant difference between simultaneous vs. staggered administration of pravastatin and BA, possibly due to a population that was heterogenous in *SLCO1B1* haplotypes.

## 1. Introduction

Hypercholesteremia is a main risk factor in cardiovascular disease. While statins are the established state-of-the-art treatment, novel cholesterol-lowering agents have been introduced in the past years. Bempedoic acid (BA) can either be administered in addition to a statin to achieve LDL cholesterol (LDL-C) goals or as (part of) an alternative therapeutic scheme in statin intolerance. BA lowers LDL-C by inhibiting adenosine triphosphate citrate lyase, an enzyme involved in an earlier step in cholesterol synthesis than 3-hydroxy-3-methyl-glutaryl-coenzyme A (HMG-CoA) reductase, which is targeted by statins [1]. The LDL-C-lowering effect of BA is moderate at approximately 25% [2,3,4]. The CLEAR outcome study has demonstrated a positive effect of BA on cardiovascular endpoints [2]. The combination of BA with a medium-dose statin has a similar LDL-C-lowering effect as monotherapy with a high statin dose, while patients may be spared the adverse effects of high-dose statin therapy [5].

The prodrug BA is well absorbed and reaches its peak concentration (C_max_) within 3.5 h [1]. In the liver, BA is either activated to the thioester conjugate ETC-1002-CoA or reversibly oxidized to a similarly active metabolite (ESP15228), which makes up approximately 20% of the circulating active compounds [6]. Both BA and ESP15228 are conjugated to inactive glucuronides by UGT2B7. Renal clearance of unchanged BA is small. The half life at steady state is 19 h ± 10 [1].

BA and its glucuronide are weak inhibitors of the transporters OATP1B1 and OATP1B3 [1], regulating intestinal uptake and the intrahepatic availability of statins. Low function of OATP1B1 is an established risk factor for myopathy under simvastatin therapy [7]. BA increases the area under the plasma concentration–time curve (AUC) of concomitant simvastatin (40 mg) 2-fold and 1.4–1.5-fold for atorvastatin (80 mg), pravastatin (80 mg), and rosuvastatin (40 mg) [1]. The extent of inhibition of OATP may be different depending on the drug concentration at the target. Therefore, the relevance of the timing of BA administration for the inhibition of OATP1B1/3 must be evaluated. In mechanistic models, the interaction between repaglinide and rifampicin via OATP1B1 (and also CYP3A4) was dependent on the timing of dosing [8]. We hypothesized that the drug interaction between BA and statins could be mitigated by staggering administration and adapting the dosing schedule.

Pravastatin is a marker substrate for hepatic uptake transport mediated by OATP1B1 and OATP1B3. The fact that pravastatin has no relevant interactions with CYP isozymes is advantageous both for clinical use and for the investigation of transporter interactions [9].

Pravastatin is an inhibitor of the HMG-CoA reductase, an important enzyme in the hepatic synthesis of cholesterol. Pravastatin is administered as the active acid. Thus, it is hydrophilic—in contrast to most other statins [10]. This makes it less likely to diffuse into the myocytes and to cross the blood–brain barrier [11]. In acidic environments, pravastatin isomerizes into 3α-hydroxy-pravastatin (pravastatin 3-iso), which makes up 10% of the excreted substance in urine to a lesser degree; also, formation of pravastatin lactone takes place [12,13]. Pravastatin 3-iso is an active isomer that inhibits HMG-CoA reductase, but much weaker than pravastatin (1/10 to 1/40) [14].

Pravastatin is a statin with long-standing use in the primary and secondary prevention of cardiovascular events; at a dose of 40 mg, its LDL-C-lowering effect is moderate, at around 25–30% [15,16,17,18]. Thus, modern goals for LDL-C reduction of ≥50% in secondary prevention [19] are most likely not met with pravastatin monotherapy.

In a clinical trial in healthy volunteers, we investigated the influence of a temporally separated administration on the drug–drug interaction between BA and pravastatin, an important OATP1B1 marker substrate.

## 2. Participants and Methods

### 2.1. Trial Design and Participants

This was a single-centre, open-label, randomized, two-arm, cross-over, phase I drug interaction trial in healthy volunteers (EudraCT-No: 2022-001096-13). The trial took place at the Pharmacological Early Clinical Trial Centre (KliPS) at Heidelberg University Hospital, Internal Medicine IX—Department of Clinical Pharmacology and Pharmacoepidemiology, from 2022 to 2023. The trial was conducted according to the guidelines of Good Clinical Practice, the ethical principles expressed in the Declaration of Helsinki, and all legal requirements for clinical trials in Germany and in the European Union. The trial was approved by the responsible Ethics Committee of the Medical Faculty of Heidelberg University (27 June 2022, AFmo-366/2022). All participants were fully informed and gave their written consent before any trial procedures.

Main inclusion criteria were age of 18–45 years, no significant findings in the medical assessment, willingness to use contraception if required (highly effective contraception in women of child-bearing potential), and participation in our genotyping biobank (ethical approval, S-026/2004, sampling of 20 mL EDTA blood). Main exclusion criteria were any clinically significant or relevant abnormalities in the medical history, physical examination, and laboratory evaluation, history of severe allergic or anaphylactic reactions, and intake of any drugs with the exception of hormonal contraception, iodine, and levothyroxine.

The primary objective of the trial was the evaluation of the OATP1B1 inhibitory effect of BA, the perpetrator drug, on the exposure with pravastatin, the victim drug, after simultaneous versus staggered administration. The primary endpoint was expressed as the geometric mean ratios (GMRs) of the AUC_∞_ and C_max_ of a single pravastatin dose of 40 mg after simultaneous compared to different schedules of staggered administration of BA 180 mg. Secondary objectives reported in the following included the evaluation of the impact of *SLCO1B1* genotypes on pravastatin pharmacokinetics (PK) after simultaneous and staggered administration and the safety and tolerability of the administered drugs.

Pravastatin was administered as a single oral dose of 40 mg. BA was administered as an oral dose of 180 mg once daily and was dosed to steady state.

**Figure 1:**
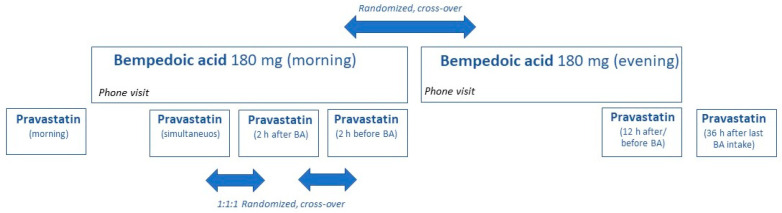
Design of randomized trial in 14 healthy participants investigating the influence of timing of oral drug administration on the effect of bempedoic acid (BA) on pravastatin. There was a baseline visit with the administration of pravastatin alone before BA was dosed to steady state. During the sequence of BA morning intake, pravastatin was administered simultaneously with BA and staggered by 2 h (before and after BA). In the sequence of BA evening intake, BA was administered 12 h and 36 h before pravastatin.

After a baseline PK evaluation with pravastatin alone, participants were started on a five-day course of daily intake of BA at home (Figure 1).

Participants were reminded by a phone call to start taking the medication and noted each drug intake in a diary. On the sixth day, PK visits with the concomitant intake of pravastatin and BA started.

Participants were individually randomized to six groups and followed a pre-defined, randomized order of visits. Half of the participants started taking BA at night and the other half took BA in the morning. There was a washout period of at least 5 days between the sequence of BA morning intake and the sequence of BA evening intake. The following dosing schedules were evaluated: simultaneous morning administration of BA and pravastatin, staggered morning administration (BA 2 h before or 2 h after administration of pravastatin), and administration of BA 12 h before the morning administration of pravastatin. In addition, we evaluated the PK of pravastatin 36 h after the termination of BA intake to investigate the duration of the interaction. At PK days, participants had to be fasting for at least 6 h. Food was served 4 h after the intake of pravastatin.

For pravastatin concentration measurements, we collected blood in lithium heparin tubes (2.7 mL) before and 0.25, 0.5, 0.75, 1, 1.25, 1.5, 1.75, 2, 2.5, 3, 3.5, 4, 5, 6, 8, 10, 12, 24, and 27 h after pravastatin administration. In addition, BA concentrations were measured after dosing to steady state to ensure adherence to treatment and prior to each following administration of pravastatin and BA at PK days (K2-EDTA, 2.7 mL).

Participants were evaluated for adverse events at each visit. Additionally, there was a laboratory assessment at three visits that included renal and liver function tests for safety reasons and measurements of uric acid, total cholesterol and LDL-C.

### 2.2. Quantification of Pravastatin, Pravastatin Lactone, and 3α-Hydroxy-Pravastatin

Pravastatin, pravastatin lactone, 3α-hydroxy-pravastatin, pravastatin-d9, and pravastatin-lactone-d9 were obtained from Santa Cruz Biotechnology (Heidelberg, Germany). Plasma concentrations were determined using a validated ultra-high performance liquid chromatography coupled with tandem mass spectrometry (UPLC-MS/MS) assay with a Waters Acquity Classic UPLC system coupled to a Waters TQ-S triple quadrupole mass spectrometer (Eschborn, Germany).

Working solutions of all analytes were prepared in a 1/1 mixture of acetonitrile and aqueous ammonium acetate buffer pH 4.5 (1 M) to impede interchange of acid and lactone species. Similarly, before analysis, plasma samples were buffered with ammonium acetate buffer pH 4.5. After addition of internal standards pravastatin-d9 and pravastatin lactone-d9, pravastatin and its metabolites were extracted from plasma using protein precipitation with 2 volume equivalents of methanol and subsequent centrifugation (16,100× *g*). The supernatants were transferred to a 96-well plate and evaporated to dryness (40 °C/15 min) using a blowdown evaporator (Ultravap; Porvair Sciences, Wrexham, Wales, UK) operated with nitrogen. Residues were diluted with 50 µL water for direct injection onto the UPLC-MS/MS system. Chromatographic separation was performed using a flow rate of 0.5 mL/min on a Waters Acquity BEH C18 column (130 Å, 1.7 μm, 2.1 × 50 mm) maintained at 40 °C with a 2 min gradient elution from 10%/90% to 60%/40% of acetonitrile/5% aqueous acetonitrile, both containing 0.1% formic acid.

Mass spectrometric quantification was performed by multiple reaction monitoring in the positive ion mode, using collision-induced dissociation. The mass spectrometer was set to a source temperature of 150 °C, a capillary voltage of 0.5 kV, a cone voltage of 10 V, desolvation gas flow (N_2_) of 1000 L/h, desolvation temperature of 600 °C, and collision gas flow (Ar) of 0.15 mL/min. Monitored mass transitions were *m*/*z* 447.5 → 327.2 for pravastatin and 3α-hydroxy-pravastatin with a collision energy of 16 V, which were chromatographically separated, *m*/*z* 429.4 → 309.2 for pravastatin lactone with a collision energy of 22 V, *m*/*z* 456.1 → 327.2 for pravastatin-d9 (16 V), and *m*/*z* 438.4 → 309.2 for pravastatin lactone-d9 (22 V).

The assay was validated in compliance with the ICH M10 guideline for bioanalytical method validation [20] in the range of 0.01 to 10 ng/mL for pravastatin lactone and 0.1 to 100 ng/mL for pravastatin and 3α-hydroxy-pravastatin using peak area ratios of analytes and internal standard. Selectivity was demonstrated by the absence of interfering peaks in 6 individual blank plasma samples. The regression coefficient of the calibration curves using 1/x^2^ weighting for linear regression was always >0.99. Intraday and interday accuracy was between 89.0% and 111.8% with corresponding precision ≤ 12.0%. Internal standard-normalized matrix effects of 6 individual plasma lots were all within 100 ± 15%.

### 2.3. Quantification of BA

BA was obtained from Toronto Research Chemicals (Toronto, ON, Canada). ESP 15228, Bempedoic Acid-D5, and ESP 15228-D4 were purchased from Clearsynth (Mumbai, India). Plasma concentrations were determined using a validated ultra-high performance liquid chromatography coupled with tandem mass spectrometry (UPLC-MS/MS) assay with a Waters Acquity I-class UPLC system coupled to a Waters TQ-S triple quadrupole mass spectrometer (Eschborn, Germany).

After addition of internal standards, BA and its metabolite were extracted from plasma using protein precipitation with 2 volume equivalents of methanol and subsequent centrifugation (16,100× *g*). A volume of 100 µL of the supernatants was transferred to a 96-well plate and diluted with 150 µL water for direct injection onto the UPLC-MS/MS system. Chromatographic separation was performed using a flow rate of 0.5 mL/min on a Waters Acquity BEH C18 column (130 Å, 1.7 μm, 2.1 × 50 mm) maintained at 60 °C with a 2 min gradient elution from 60%/40% to 65%/35% of methanol/5% aqueous acetonitrile, both containing 0.1% formic acid.

Mass spectrometric quantification was performed by multiple reaction monitoring in the negative ion mode, using collision-induced dissociation. The mass spectrometer was set to a source temperature of 150 °C, a capillary voltage of 1.5 kV, a cone voltage of 40 V, desolvation gas flow (N_2_) of 1000 L/h, desolvation temperature of 600 °C, and collision gas flow (Ar) of 0.15 mL/min. Monitored mass transitions were *m*/*z* 343.2 → 299.2 for BA (*m*/*z* 348.2 → 304.2 for bempedoic acid-D5) at a collision energy of 28 V and *m*/*z* 341.2 → 297.2 for ESP15228 (*m*/*z* 345.2 → 301.2 for ESP15228-D4) at a collision energy of 26 V.

The assay was validated in compliance with the ICH M10 guideline for bioanalytical method validation [20] in the range of 0.05 to 50 µg/mL for both analytes using peak area ratios of analytes and internal standard. Selectivity was demonstrated by the absence of interfering peaks in 6 individual blank plasma samples. The regression coefficient of the calibration curves using 1/x^2^ weighting for linear regression was always >0.99. Intraday and interday accuracy ranged between 90.5% and 113.9% with corresponding precision ≤ 10.5%. Internal standard-normalized matrix effects of 6 individual plasma lots were all within 100 ± 15%.

### 2.4. Genotyping of SLCO1B1

The two single nucleotide polymorphisms rs2306283 (c.388A>G) and rs4149056 (c.521T>C) in the *SLCO1B1* gene were determined using the hybridization probes format on the LightCycler 480^®^ (Roche Applied Sciences, Mannheim, Germany) as published previously [21].

### 2.5. Pharmacokinetic and Statistical Analysis

Outcome measures were the AUC, extrapolated to infinity (AUC_∞_), and C_max_ of pravastatin, pravastatin 3-iso, and pravastatin lactone. Non-compartmental PK analysis was performed with Phoenix WinNonlin Version 8.4 (Certara, Princeton, NJ, USA). Descriptive statistics included the geometric mean of AUC_∞_ and C_max_ and the arithmetic mean of t_max_ with a 95% confidence interval (CI). For the calculation of geometric means, values of zero were excluded. Log-transformed pravastatin AUC_∞_ and C_max_ were compared with one-way ANOVA (mixed-effects model in case of missing values) with 90% CI, comparing all visits to every other visit. The CI was back-transformed into the desired CI for the GMR [22]. The primary objective was analyzed in all participants who completed all pravastatin PK visits. The secondary objective was evaluated in all haplotype sub-groups with n ≥ 2 and at least 2 fully completed pravastatin PK visits. Two-way ANOVA was applied to evaluate the impact of haplotype, study visit, and sex. The relationship between BA and pravastatin concentrations was evaluated with Pearson correlation coefficients. The statistical analysis was conducted in GraphPad Prism Version 10 (La Jolla, CA, USA). A *p* value < 0.05 was considered significant.

## 3. Results

### 3.1. Study Population

Seventeen participants (9 females) with a mean age of 26 ± 3.9 years and a mean body mass index of 23 ± 2.4 kg/m^2^ were included. All were genotyped (Table 1), and in all participants, the baseline PK evaluation with pravastatin was completed. Three participants terminated the trial prematurely unrelated to study medication. Fourteen participants completed all trial visits (per-protocol population). Due to difficulties with recruitment, the per-protocol population included 10 instead of the planned 12 participants with *1/*1 or *1/*37 haplotypes.

### 3.2. Pravastatin Exposure

The exposure of pravastatin, 3α-hydroxy-pravastatin (pravastatin 3-iso), and pravastatin lactone was compared between all visits in the per-protocol population (Table 2).

**Figure 2:**
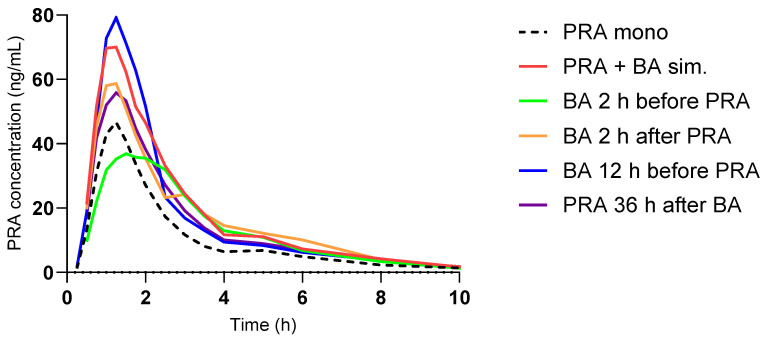
Plasma exposure of pravastatin (PRA; geometric mean) after oral administration of 40 mg pravastatin with no (PRA mono), simultaneous (sim.), and staggered oral co-administration of 180 mg bempedoic (BA). For the calculation of the geometric means, concentrations of zero were excluded.

After simultaneous administration of pravastatin and BA, the AUC_∞_ of pravastatin was 180% and was 195% for C_max_ compared to pravastatin alone (Figure 2). There were no statistically significant differences in pravastatin 3-iso or pravastatin lactone between any pravastatin visits.

### 3.3. Bempedoic Acid

**Figure 3:**
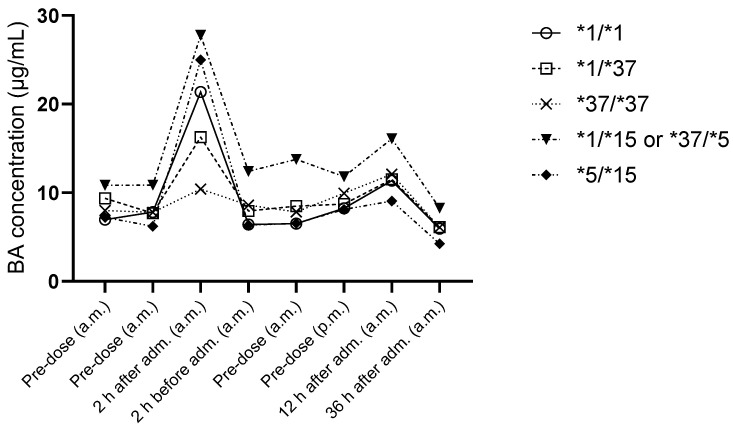
Plasma bempedoic acid (BA) concentrations throughout the trial according to *SLCO1B1* haplotype (*n* = 14).

In the per-protocol population, plasma trough concentrations of BA at steady state ranged between 7.88 µg/mL (95% CI 6.47–9.60 µg/mL) and 19.0 µg/mL (95% CI 15.0–23.9 µg/mL; 2 h after BA administration = prior to staggered pravastatin administration). Two hours after administration, the participant carrying the *37/*37 or *1/*37 haplotypes showed a significantly lower concentration than the participants carrying the *1/*15 or *37/*5, *5/*15, or *15/*15 haplotypes (Figure 3). The average ratio of the active metabolite to BA ranged from 16% (2 h after administration) to 34% (2 h before administration, pre-dose a.m., and 36 h after administration). There was no correlation between the concentration of BA and the corresponding exposure with pravastatin for any study visit.

### 3.4. LDL-C-Lowering Effect

LDL-C at baseline was 98.4 mg/dL ± 27.2 (mean ± SD) in the per-protocol population. LDL-C decreased by 26.8 mg/dL (95% CI 14.0–39.6) at BA steady state. After the additional intake of three single doses of 40 mg pravastatin with BA continued, the difference to baseline increased to 32.9 mg/dL (95% CI 18.3–47.6).

### 3.5. Evaluation of the Impact of SLCO1B1 Haplotypes

**Figure 4:**
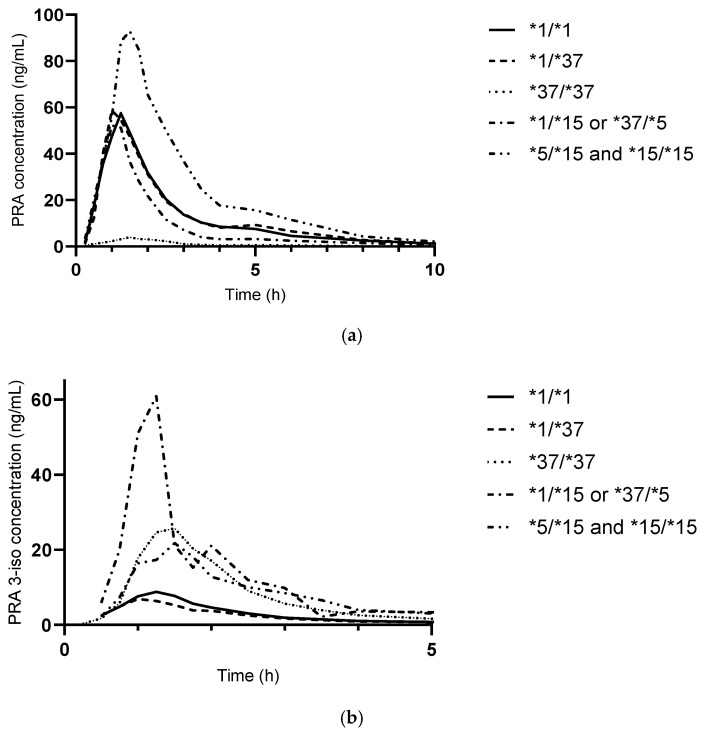
Association of *SLCO1B1* haplotypes with pravastatin (PRA) exposure (**a**) and 3α-hydroxy-pravastatin (PRA 3-iso) (**b**) (geometric mean) after administration of 40 mg pravastatin (alone) (*n* = 17).

At baseline, variation in the AUC_∞_ of pravastatin was explained to 79% by *SLCO1B1* haplotype and to 9% by sex (but not by weight, grouped by less than and at least 70 kg). The participant carrying the *37/*37 haplotype showed significantly lower pravastatin exposure compared to all other haplotypes (AUC_∞_ 6.75% of the *1/*1 AUC_∞_; 7.53% of the AUC_∞_ of all), and the relation with haplotype was not significant anymore when excluding this participant (Figure 4).

For the AUC_∞_ of pravastatin and pravastatin 3-iso, haplotype was a significant source of variation across all study visits (63% and 20%, respectively), while the study visit had no significant impact.

**Figure 5:**
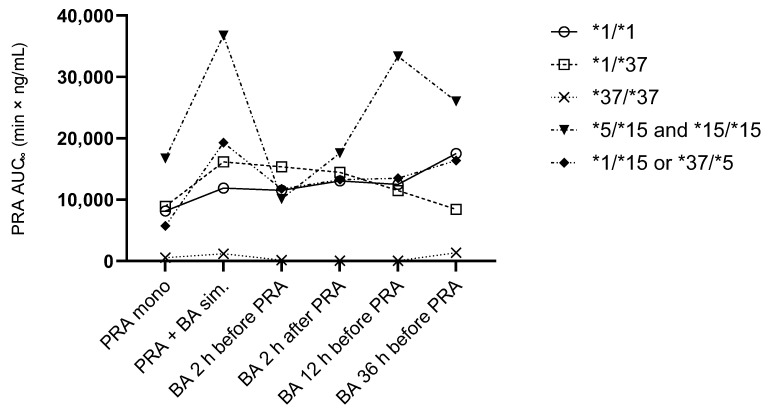
Pravastatin AUC_∞_ per haplotype at baseline (PRA mono), with simultaneous (sim.) administration of 40 mg pravastatin (PRA) and 180 mg bempedoic acid (BA) and with different schedules of staggered co-administration (*n* = 17). Geometric mean. AUC_∞_: area under the time–concentration curve extrapolated to infinity.

**Figure 6:**
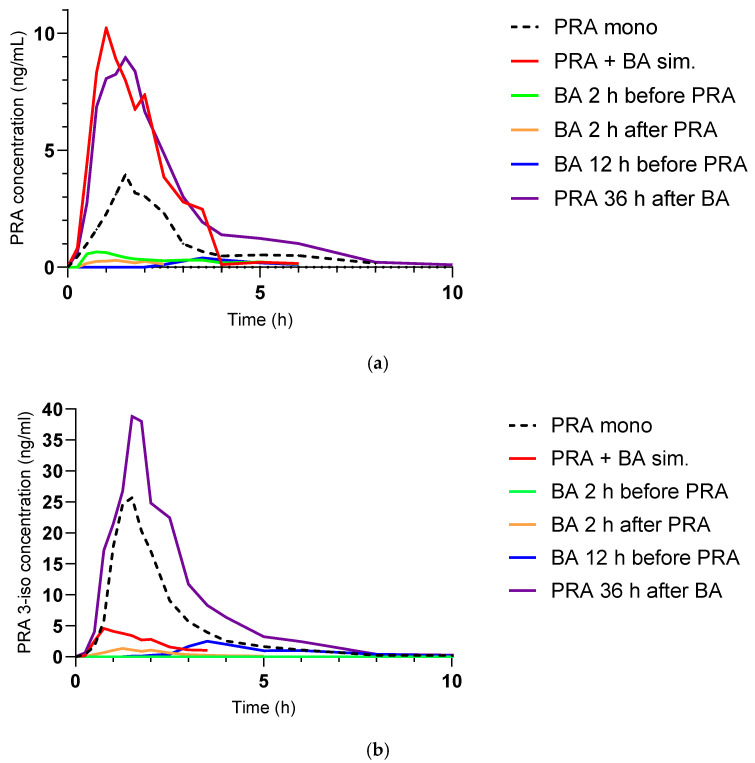
Pravastatin (PRA) (**a**) and 3α-hydroxy-pravastatin (PRA 3-iso) (**b**) exposure after administration of 40 mg pravastatin only (PRA mono) and with simultaneous (sim.) or staggered co-administration of 180 mg bempedoic acid (BA) in the participant with the *37/*37 haplotype.

The AUC_∞_ per haplotype subgroup is shown across all study visits in Figure 5. In the subgroup carrying the *1/*1 haplotype, the AUC_∞_ of pravastatin with simultaneous administration of BA, 12 h after BA administration, and 36 h after last BA administration was significantly different from the administration of pravastatin only (GMR 1.46, 90% CI 1.12–1.70; GMR 1.53, 90% CI 1.13–2.08; GMR 2.15, 90% CI 1.01–4.07, respectively). There was no significant difference in the AUC_∞_ of pravastatin between the simultaneous administration of BA and the administration of pravastatin only in the subgroup with *1/*37 and in the subgroup with *1/*15 or *37/*5 haplotypes. The subgroup of *15/*15 and *5/*5 did not contain enough data for statistical analysis. The participant carrying the *37/*37 haplotype showed visibly lower pravastatin exposure with any staggered administration of pravastatin and BA than with simultaneous administration and also after the end of BA administration (Figure 6).

### 3.6. Adverse Events

All adverse events were transient (most frequently reported: headache, abdominal symptoms, and deviations in laboratory values). They were mild or moderate in intensity, except for syncopes following blood sampling, and an increase in creatine kinase that was not related to study medication.

## 4. Discussion

As expected [1], pravastatin exposure significantly increased after simultaneous administration of 40 mg pravastatin with BA and this increase was larger than the 1.4–1.5-fold change described in the SmPC for the higher dose of 80 mg pravastatin [1]. There was no significant difference in pravastatin exposure after staggered administration either when compared to simultaneous administration or to the baseline administration of pravastatin alone. Even though not statistically significant, the PK parameters under the schedules of staggered administration seemed similar to each other and consistently lower than with simultaneous administration. It cannot be excluded that this trend did not achieve statistical significance due to a small sample size and heterogeneity in *SLCO1B1* haplotypes.

The *SLCO1B1* c.521T>C variant has a prevalence of 15% in Caucasians [26,27] and leads to increased systemic exposure of pravastatin [28]. In addition, this variant has been associated with increased pravastatin 3-iso exposure [11], which is not only an active metabolite but also inhibits OATP1B1-mediated uptake of pravastatin. In OATP1B1*5 and *15 transfected cells, IC_50_ values for pravastatin 3-iso were lower than for *1 and *37 [29]. Thus, OATP1B1-inhibtion by pravastatin 3-iso could have interfered with the effect of BA especially in those participants carrying *5/*15, *15/*15, or *1/*15 or *37/*5 haplotypes; however, this group was small and not all of those participants completed all study visits, so analysis was limited.

The only participant with the *37/*37 haplotype showed low pravastatin exposure. Indeed, the *37 haplotype has been associated with a decreased AUC of pravastatin in clinical trials, but evidence from PK trials is not fully conclusive [24,25]. In line with a possibly increased hepatic uptake is the finding that the c.388A>G variant has been associated with an (small) improved LDL-C-lowering effect [26]. Interestingly, with all staggered administration schedules of BA, the participant with the 37/*37 haplotype showed very low pravastatin exposure, while with simultaneous administration, as well as after the end of BA administration, the AUC_∞_ was comparably higher.

A decreased OAPT1B1 function because of the *SLCO1B1* c.521T>C variant leads to increased systemic and reduced hepatic statin concentrations; it is strongly related to the risk of myopathy (odds ratio of 4.5 per copy of C allele), and—to a lesser extent—to the LDL-C-lowering effect [26]. However, there was no impact of the *SLCO1B1* c.521T>C variant on the LDL-C lowering in pravastatin in a small trial in healthy volunteers [30]. Parallel effects might be expected with drug-induced OATP1B1 inhibition. In the phase III trials with BA that accepted a maximum tolerated statin dose as concomitant therapy, there were signals that patients on BA reported more pain in the extremities, but myalgia was not clearly more frequent in patients with BA and statins compared to those without BA [31,32,33]. This indicates that the clinical impact of this interaction might not be finally evaluated yet. Also, it is unclear which statins were used and if this observation also represents pravastatin. In addition, rare *SLCO1B1* haplotypes might have been uncommon in those study populations. Prospectively, the extent of potential adverse effects from increased exposure with statins interacting with BA might be better understood after the analysis of post-approval real-world data in large populations.

There is some evidence on the impact of staggered administration on drug interactions. A clinical trial with simvastatin and fenofibrate did not show any impact of staggered as opposed to simultaneous administration, with the mechanism of this interaction remaining unclear [34]. Another clinical trial, focusing on an interaction at the level of absorption, found that the interaction between cephalexin and zinc could be mostly avoided by administering zinc 3 h after cephalexin [35]. We are not aware of any previous clinical trials on the impact of the timing of dosing on drug interaction at the level of OATP1B1.

The study was limited by the sample size, which was also the result of recruitment difficulties during the COVID-19 period. Different OATP1B1 haplotypes were included intentionally, but in consequence, the resulting heterogeneity of the trial population and the rather small number of participants with normal OATP1B1 function limited the evaluation of the primary objective. We cannot exclude that the timing of BA dosing might have a statistically significant impact on pravastatin exposure in a larger or more homogenous cohort.

The only participant with the *37*37 haplotype showed much lower pravastatin exposure than all other haplotypes. For this reason, the PK data for this participant had a relevant impact on the evaluation of the significance of haplotypes. Low exposure associated with *37 has been described before, but it was only lower by 35% [24,25]. We cannot fully exclude that there was possibly another factor related to the very low exposure in this single participant.

Furthermore, it is not clear whether the findings of this trial can be transferred to the case of other statins that are OATP1B1 substrates. Research on the interaction between BA and statins that have different properties would be of interest.

In conclusion, pravastatin exposure was increased at BA steady state but was not significantly different between simultaneous and staggered co-administration of BA, while it varied according to *SLCO1B1* haplotype.

## Figures and Tables

**Table 1:** *SLCO1B1* genotypes of the participants.

rs2306283	rs4149056	Haplotypes	*SLCO1B1* Phenotype ^(1)^	No. of Participants (Per-Protocol Population)
AA	TT	*1/*1	Normal function	5 (4)
AG	TT	*1/*37	Normal function ^(2)^	7 (6)
GG	TT	*37/*37	Normal function ^(2)^	1
AG	TC	*1/*15 or *37/*5	Intermediate function	2
AG	CC	*5/*15	Low function	1
GG	CC	*15/*15	Low function	1 (0)

^(1)^ Assignment of likely *SLCO1B1* phenotype according to Ramsey and co-workers [7,23]. ^(2)^ Haplotype * 37 (= *1B in previous nomenclature [23]) is possibly associated with a decreased AUC of pravastatin [24,25].

**Table 2:** Exposure after simultaneous (sim.) administration of 40 mg pravastatin (PRA) and 180 mg bempedoic acid (BA) in relation to the baseline evaluation with pravastatin alone (PRA mono) and in relation to different schedules of staggered co-administration.

		PRA Mono	PRA + BA sim.	BA 2 h Before PRA	BA 2 h After PRA	BA 12 h Before PRA	BA 36 h Before PRA
PRA	AUC_∞_ (min*ng/mL)	7303 (4302–12,398)	13,123 (7512–22,926)	9496 (4620–19,519)	9252 (3545–24,149)	9241 (3773–22,638)	10,854 (5293–22,257)
	GMR (90% CI)	1.80 (1.31–2.46)		1.38 (0.82–2.33)	1.42 (0.66–3.06)	1.42 (0.70–2.87)	1.21 (0.69–2.10)
	C_max_ (ng/mL)	55.7 (31.4–98.6)	108 (59.5–198)	61.7 (26.9–141)	66.5 (24.7–179)	72.2 (27.7–188)	85.4 (41.3–176)
	GMR (90% CI)	1.95 1.40–2.72		1.76 (0.92–3.37)	1.63 (0.72–3.69)	1.50 (0.65–3.49)	1.27 (0.75–2.16)
	T_max_ (min)	67.9 (58.5–77.4)	67.7 (46.5–88.9)	85.9 (62.7–109)	69.7 (59.8–79.6)	81.5 (57.5–106)	66.5 (50.0–83.0)
PRA 3-iso	AUC_∞_ (min*ng/mL)	1746 (732–4162)	2394 (1322–4335)	*3720* (*1706*–*8110*)	3762 (1972–7178)	3863 (2387–6251)	2822 (1547–5146)
	GMR (90% CI)	1.37 (0.35–5.33)		*0.64* (*0.20*–*2.54*)	0.64 (0.30–1.35)	0.62 (0.29–1.32)	0.85 (0.34–2.09)
	C_max_ (ng/mL)	15.3 (5.96–39.1)	23.4 (12.7–43.1)	*31.0* (*12.7*–*76.1*)	27.6 (12.4–61.6)	32.5 (19.2–55.1)	24.6 (13.5–45.1)
	GMR (90% CI)	1.53 (0.36–6.51)		*0.75* (*0.21*–*3.43*)	0.85 (0.30–2.41)	0.72 (0.32–1.63)	0.95 (0.41–2.18)
	T_max_ (min)	66.6 (56.1–77.1)	69.8 (51.0–88.6)	85.8 (59.4–112)	77.1 (58.3–96.0)	85.9 (55.8–116)	65.4 (49.3–81.6)
PRA lactone	AUC_∞_ (min*ng/mL)	120 (68.8–209)	154 (81–291)	*164* (*116*–*233*)	*175* (*107*–*288*)	*181* (*108*–*303*)	149 (65.8–339)
	GMR (90% CI)	1.28 (0.98–1.68)		*0.94* (*0.78*–*1.67*)	*0.88* (*0.71*–*1.61*)	*0.85* (*0.72*–*1.47*)	1.03 (0.54–1.97)
	C_max_ (ng/mL)	(0.39–1.51)	1.03 (0.50–2.13)	*1.05* (*0.72*–*1.53*)	*1.12* (*0.67*–*1.86*)	*1.18* (*0.70*–*2.0*)	0.95 (0.43–2.09)
	GMR (90% CI)	1.34 (0.91–1.97)		*0.98* (*0.80*–*1.91*)	*0.92* (*0.77*–*1.73*)	*0.87* (*0.75*–*1.60*)	1.09 (0.54–2.19)
	T_max_ (min)	69.1 (57.5–80.7)	67.6 (50.7–84.6)	81.4 (57.5–105)	60.1 (45.3–74.9)	75.1 (44.9–105)	62.3 (48.8–75.8)

Geometric mean (AUC_∞_ and C_max_) and mean (T_max_) with 95% confidence interval (CI). Zero values were excluded to calculate the geometric mean (in *italics*). AUC = area under the concentration–time curve, GMR = geometric mean ratio, PRA = pravastatin, PRA 3-iso = 3α-hydroxy-pravastatin.

## Data Availability

The data presented in this study are available from the corresponding author on reasonable request. The data are not publicly available due to legal reasons.
